# Supplementary material for: Microbial Profiling of Potato-Associated Rhizosphere Bacteria under Bacteriophage Therapy
Source: Antibiotics (Basel). 2022 Aug 18;11(8):1117. doi: 10.3390/antibiotics11081117 (PMC9405460; doi:10.3390/antibiotics11081117)
Supplement: Supplementary file 1 [file antibiotics-11-01117-s001.zip › antibiotics-1829310-supplementary.pdf]

Table S1. Some characteristics of the experimental soil and cattle manure (CM)

Characteristics	Soil	CM
<u>Particle size distribution (%):</u>		
Textural grade	Sand	-
pH	8.01†	7.39‡
EC_e (dS m^{-1})§	1.11	10.7
<u>Soluble cations (meq l^{-1})</u> §		
Na^{+}	3.8	12.0
K^{+}	1. 54	9.91
Ca^{2+}	21.4	26.2
Mg^{2+}	9.3	6.0
<u>Soluble anions (meq l^{-1})</u> §		
Cl^{-}	0.16	1.38
HCO_3^{-}	2.20	36.0
SO_4^{2-}	0.43	5.98
CO_3^{2-}	0.00	0.0
Organic C (g kg^{-1})	0.36	12.6
Total N (g kg^{-1})	0.21	11.6
Available N (mg kg^{-1})	1.29	79.7
Available P (mg Kg^{-1})	3.0	101

† In soil-water suspension (1:2.5)
‡ In CM-water suspension (1:5)
§ In soil and CM saturated extract Statistical analysis

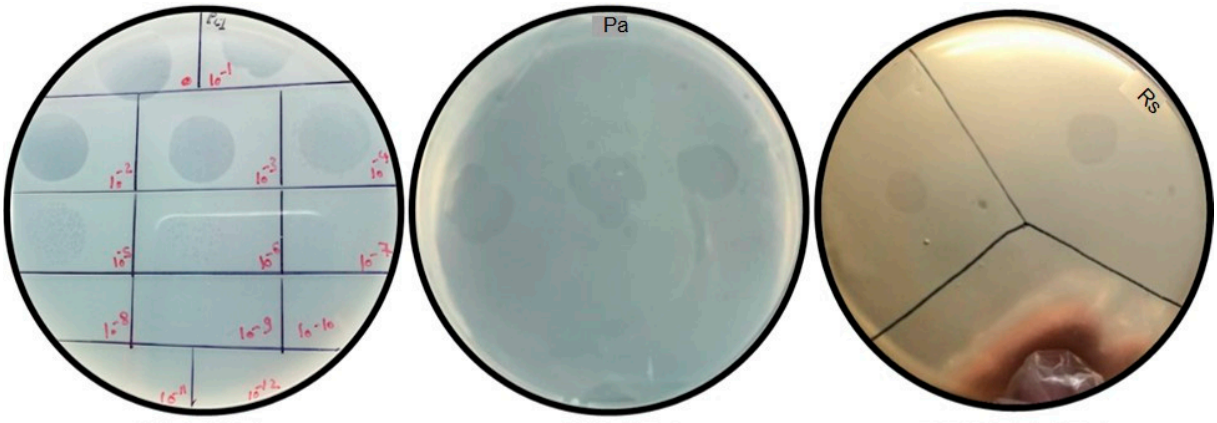

Wc4 (SPc) CX5 (SPa) P-PSG-11 (SRs)

Figure S1. Phage plaque assay

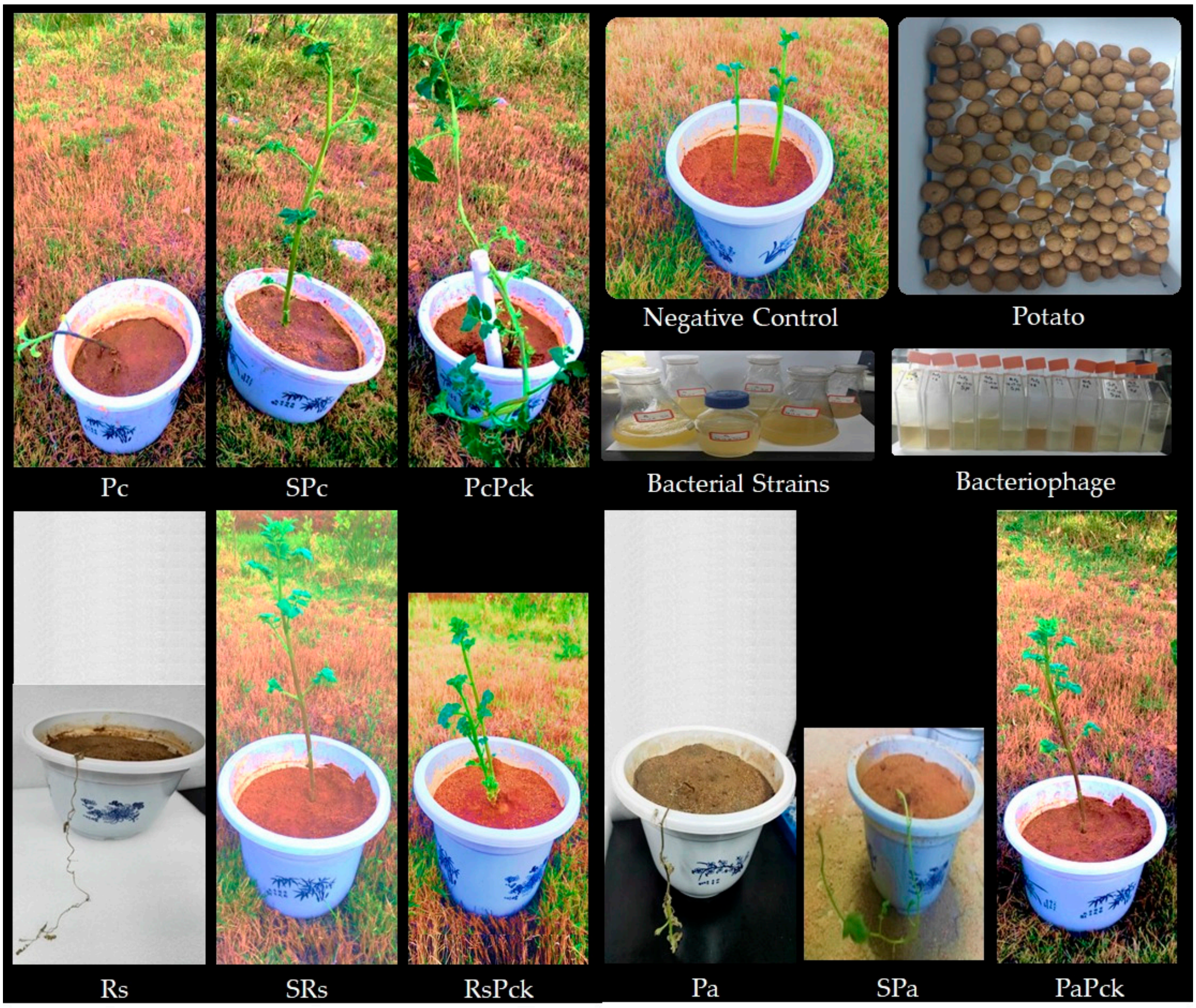

Figure S2. Phage biocontrol experiment
